# Supplementary material for: Hybrid Models and Biological Model Reduction with PyDSTool
Source: PLoS Comput Biol. 2012 Aug 9;8(8):e1002628. doi: 10.1371/journal.pcbi.1002628 (PMC3415397; doi:10.1371/journal.pcbi.1002628)
Supplement: Text S4 — Complete source code for the PyDSTool package (version 0.88.120504). Includes API documentation and help files linking to web pages. This file is identical to the current public release on Sourceforge.net. (ZIP) [file pcbi.1002628.s004.zip › PyDSTool/html/PyDSTool.Toolbox.optimizers.line_search.damped_line_search-pysrc.html]

xml version="1.0" encoding="ascii"?


PyDSTool.Toolbox.optimizers.line\_search.damped\_line\_search


| Home | Trees | Indices | Help | PyDSTool |
| --- | --- | --- | --- | --- |

Package PyDSTool ::
Package Toolbox ::
Package optimizers ::
Package line\_search ::
Module damped\_line\_search

[hide private]

[frames] | no frames]

# Source Code for Module PyDSTool.Toolbox.optimizers.line\_search.damped\_line\_search

```
 1   
 2  # Matthieu Brucher 
 3  # Last Change : 2007-08-26 19:47 
 4   
 5  """ 
 6  A damped line search 
 7  """ 
 8   


9 -class DampedLineSearch(object):


10    """ 
11    A damped line search, takes a point and a direction. 
12    Tests a new point for minimization, if it is greater that the current cost times (1 + error), 
13      the step is divided by two, until the step is too small 
14    """ 


15 -  def __init__(self, min_alpha_step, damped_error, alpha_step = 1., **kwargs):


16      """ 
17      Needs to have : 
18        - a minimum step size (min_alpha_step) 
19        - a factor to allow the cost to rise a little bit (damped_error) 
20      Can have : 
21        - a step modifier, a factor to modulate the step (alpha_step = 1.) 
22      """ 
23      self.minStepSize = min_alpha_step 
24      self.dampedError = damped_error 
25      self.stepSize = alpha_step

26   


27 -  def __call__(self, origin, function, state, **kwargs):


28      """ 
29      Returns a good candidate 
30      Parameters : 
31        - origin is the origin of the search 
32        - function is the function to minimize 
33        - state is the state of the optimizer 
34      """ 
35      direction = state['direction'] 
36      if 'initial_alpha_step' in state: 
37        stepSize = state['initial_alpha_step'] 
38      else: 
39        stepSize = self.stepSize 
40      currentValue = function(origin) 
41      optimalPoint = origin + stepSize * direction 
42      newValue = function(optimalPoint) 
43   
44      while(newValue > currentValue * (1. + self.dampedError)): 
45        stepSize /= 2. 
46        if stepSize < self.minStepSize: 
47          break 
48        optimalPoint = origin + stepSize * direction 
49        newValue = function(optimalPoint) 
50      else: 
51        state['alpha_step'] = stepSize 
52        return optimalPoint 
53      state['alpha_step'] = 0. 
54      return origin

55
```

  


| Home | Trees | Indices | Help | PyDSTool |
| --- | --- | --- | --- | --- |

|  |  |
| --- | --- |
| Generated by Epydoc 3.0.1 on Fri May 4 15:24:18 2012 | http://epydoc.sourceforge.net |
